# A Reliable and Accessible Caregiving Language Model (CaLM) to Support Tools for Caregivers: Development and Evaluation Study

**DOI:** 10.2196/54633

**Published:** 2024-07-31

**Authors:** Bambang Parmanto, Bayu Aryoyudanta, Timothius Wilbert Soekinto, I Made Agus Setiawan, Yuhan Wang, Haomin Hu, Andi Saptono, Yong Kyung Choi

**Affiliations:** 1 Department of Health Information Management University of Pittsburgh Pittsburgh, PA United States

**Keywords:** large language model, caregiving, caregiver, informal care, carer, GPT, language model, LLM, elderly, aging, ChatGPT, machine learning, natural language processing, NLP

## Abstract

**Background:**

In the United States, 1 in 5 adults currently serves as a family caregiver for an individual with a serious illness or disability. Unlike professional caregivers, family caregivers often assume this role without formal preparation or training. Thus, there is an urgent need to enhance the capacity of family caregivers to provide quality care. Leveraging technology as an educational tool or an adjunct to care is a promising approach that has the potential to enhance the learning and caregiving capabilities of family caregivers. Large language models (LLMs) can potentially be used as a foundation technology for supporting caregivers. An LLM can be categorized as a foundation model (FM), which is a large-scale model trained on a broad data set that can be adapted to a range of different domain tasks. Despite their potential, FMs have the critical weakness of “hallucination,” where the models generate information that can be misleading or inaccurate. Information reliability is essential when language models are deployed as front-line help tools for caregivers.

**Objective:**

This study aimed to (1) develop a reliable caregiving language model (CaLM) by using FMs and a caregiving knowledge base, (2) develop an accessible CaLM using a small FM that requires fewer computing resources, and (3) evaluate the model’s performance compared with a large FM.

**Methods:**

We developed a CaLM using the retrieval augmented generation (RAG) framework combined with FM fine-tuning for improving the quality of FM answers by grounding the model on a caregiving knowledge base. The key components of the CaLM are the caregiving knowledge base, a fine-tuned FM, and a retriever module. We used 2 small FMs as candidates for the foundation of the CaLM (LLaMA [large language model Meta AI] 2 and Falcon with 7 billion parameters) and adopted a large FM (GPT-3.5 with an estimated 175 billion parameters) as a benchmark. We developed the caregiving knowledge base by gathering various types of documents from the internet. We focused on caregivers of individuals with Alzheimer disease and related dementias. We evaluated the models’ performances using the benchmark metrics commonly used in evaluating language models and their reliability for providing accurate references with their answers.

**Results:**

The RAG framework improved the performance of all FMs used in this study across all measures. As expected, the large FM performed better than the small FMs across all metrics. Interestingly, the small fine-tuned FMs with RAG performed significantly better than GPT 3.5 across all metrics. The fine-tuned LLaMA 2 with a small FM performed better than GPT 3.5 (even with RAG) in returning references with the answers.

**Conclusions:**

The study shows that a reliable and accessible CaLM can be developed using small FMs with a knowledge base specific to the caregiving domain.

## Introduction

### Background

The number of family caregivers for people with complex and chronic conditions, such as dementia and other disabilities, is increasing dramatically. In 2020, an estimated 53 million adults in the United States (1 in 5) served as family caregivers, up from the estimated 43.5 million in 2015 [1]. These numbers are expected to climb dramatically over the next 30 years [1,2]. Unlike professional health care providers and caregivers, family caregivers often assume this role without formal education or training [3], leaving them underprepared for the complex tasks of caregiving. This lack of preparation can lead to increased stress and a sense of being overwhelmed [4]. Previous studies have shown that family caregivers are at risk for poor psychological health, physical health, and quality of life; strains in family relationships; and restrictions in social and work participation [5-8]. There is an urgent need to enhance the capacity of caregivers, although there remain unanswered questions regarding how best to support the diverse needs of family caregivers [9]. One of the key features of successful interventions in supporting caregivers is to equip caregivers with practical knowledge and skills for providing care, including knowledge about the care recipient’s condition, associated symptoms, and symptom progression, as well as skills that enable them to address the needs of the care recipient [10]. In this context, technology can play a pivotal role in supporting caregivers as a means of delivering educational tools or serving as a supplementary aid in the caregiving process [2,11-14]. Notably, 2 of the 10 research priorities identified by the Summit on Family Caregiving focus on the use of technology for family caregiving, although technology can also be used to support the remaining 8 research priorities [2].

Recent advances in generative artificial intelligence (AI) [15-19] have resulted in the popularity and widespread use of large language models (LLMs), such as ChatGPT, captivating global interest with their ability to generate intelligent and context-aware responses to a wide spectrum of users’ questions or prompts [18,20-22]. These LLMs fall into a category called foundation models (FMs) [23]. An FM is a large-scale machine-learning model trained on diverse and comprehensive data sets. These data sets equip the FMs with the versatility to perform a wide range of tasks across different domains [17,19,23]. An FM provides a base, or a foundation, on which other specific models can be built. FMs, such as OpenAI’s GPT-3 and GPT-4, are pretrained using diverse corpora of the content found across the internet. These pretrained models can serve as the basis for developing educational content as well as interactive agents, such as chatbots, to support caregivers [24]. These interactive agents will be able to address common requests from caregivers, including answering questions about the care recipient’s condition, associated symptoms, and symptom progression, as well as teaching skills that enable caregivers to address the needs of the care recipient [25].

However, FMs often fail to answer domain-specific questions that require factual knowledge. The responses generated by these models, while impressive and convincing, can be misleading or completely wrong—a phenomenon called hallucination [26]. This issue is particularly problematic because it may be inherent to LLMs even when the size gets larger, and it is a feature, not a bug [26,27]. This means that the system cannot be fully trusted in contexts where accuracy is paramount, such as in caregiving. In such contexts, the reliability and factual accuracy of information are nonnegotiable, as they underpin decisions that have direct consequences on the health and well-being of individuals. Furthermore, aside from hallucination, even the most powerful pretrained FMs will most likely not meet the specific needs of caregivers right out of the box.

Adaptations have been developed to equip FMs to meet the specific needs of particular tasks, and 3 of the most prominent adaptation methods are prompt engineering, fine-tuning, and, most recently, retrieval augmented generation (RAG) [28-30]. Prompt engineering is the most popular method, and its goal is to guide the model toward desirable answers. This is the simplest approach because it does not involve retraining the FM or developing a knowledge base. RAG, on the other hand, introduces additional layers that use external knowledge (data sources) to provide the context for improving the performance and relevance of the FM. It is more complex to implement than prompt engineering because it requires the development of domain knowledge. Fine-tuning is the most complex method in terms of implementation because it requires retraining the FM. By strategically combining these methods, it is possible to enhance an FM’s functionality, tailoring it to deliver more precise and useful outputs for domain-specific applications such as caregiving.

### Objectives

The objective of this exploratory study was to develop a reliable and accessible caregiving language model (CaLM). To achieve reliability, the CaLM will use the RAG framework that employs a caregiving knowledge base to generate prompts to provide a caregiving context to any questions from users. The CaLM is further fine-tuned by retraining the FM with caregiving-related data that can train the FM to provide authoritative references and informed answers to caregiving-related questions. To achieve the goal of accessibility, the CaLM uses a small FM that can be deployed with a modest computing infrastructure in a home or a small organization. The CaLM can further be used to develop downstream technologies, such as a caregiving chatbot, aiming to support caregivers in various settings.

## Methods

### CaLM Architecture

**Figure 1:**
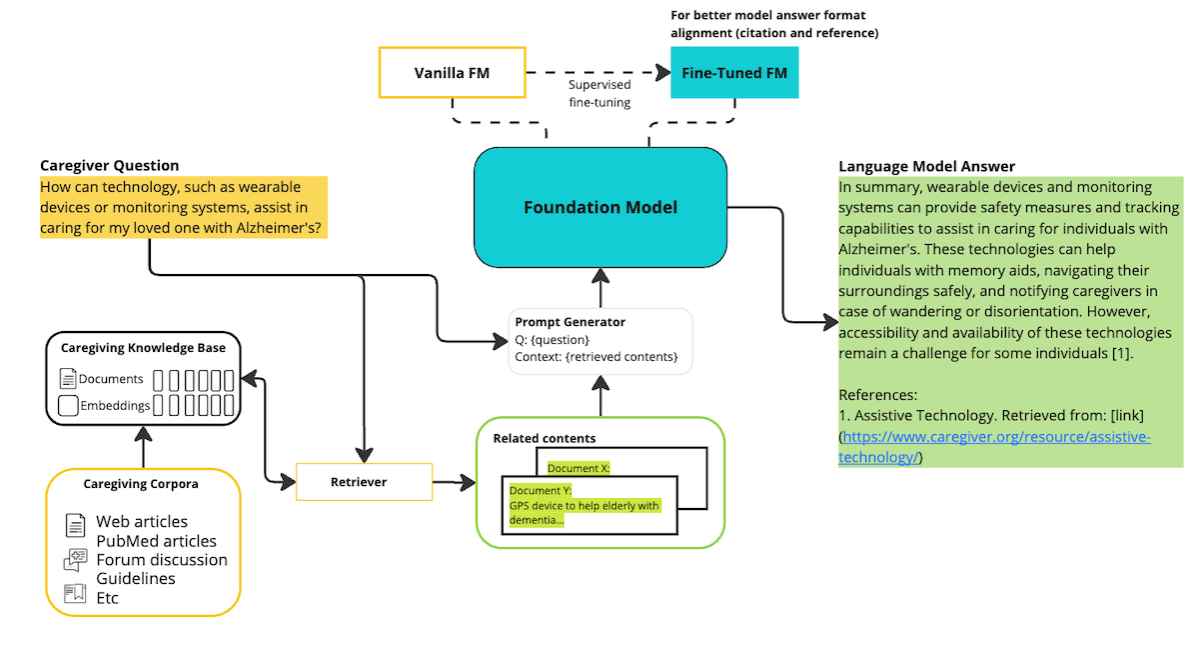
Caregiving language model architecture. FM: foundation model.

The overall architecture of the CaLM is illustrated in Figure 1. The key components of the CaLM are the caregiving knowledge base, a fine-tuned FM, and a retriever module. The knowledge base and retriever module are part of the RAG framework [28]. An interaction system can be added if the CaLM is implemented as a conversation agent such as a chatbot. The CaLM uses a RAG framework to give the FM access to information that is specific to caregiving, and it uses fine-tuning to further retrain the FM to answer questions related to caregiving. Each of the modules will be discussed further in this section.

The most common interaction between caregivers and language models is an open-ended question and answer (Q-A) method [28]. In a regular FM, which we call a vanilla FM, a question from caregivers is submitted to the FM, and the FM retrieves answers based on its pretrained knowledge representation. In the CaLM, a question from a user is appended by a prompt generated from the caregiving knowledge base before it is submitted to the FM. The retriever module in the CaLM retrieves semantically similar information from the caregiving knowledge base and creates prompts to accompany the user question. The FM uses the question and the prompts to get a more relevant answer than it could without the prompts.

In the CaLM, the FM is further fine-tuned by retraining the FM using a Q-A training set containing common questions and answers related to caregiving. Fine-tuning can be used to impart FMs with domain-specific terminology and with personalization for a specific population. This technique can be used to train FMs in areas important to caregiving, such as empathy. The question from the caregiver, combined with prompts from RAG, is submitted to the fine-tuned FM, which subsequently provides answers that are more accurate and more knowledgeable regarding caregiving.

### FM Description

LLMs, such as ChatGPT, are subsets of AI systems called FMs. FMs are designed to be general purpose models capable of performing many different tasks and can also be adapted to a variety of tasks. As the name implies, we will use FMs as the base foundation for the CaLM. Hundreds of FMs are currently available, and a recent paper catalogs more than 75 major transformer-based models alone [31]. The goal of this project is to build a reliable and accessible model in the caregiving domain; therefore, the FM needs to fulfill 2 requirements: good performance and relatively small size. In this project, we have evaluated 3 different FMs, which are summarized in Table 1. Falcon and LLaMA (large language model Meta AI) 2 were chosen because the models were at the top of the Open LLM Leaderboard [32] when this study was carried out. LLaMA [33] is a family of LLMs released by Meta starting in February 2023. Falcon is a family of LLMs developed by the Technology Innovation Institute (TII) based in Abu Dhabi, United Arab Emirates [34].

**Table 1:** Foundation models used in the study.

Foundation model	Developer	Description	Licensing	Parameters
LLaMA^a^ 2 7B	Meta	Large language model of Meta AI; top-ranked model	Open source	7 billion
Falcon 7B	Technology Innovation Institute, UAE	Part of the Falcon LLM^b^ family; Apache permissive license; top-ranked model	Open source	7 billion
GPT 3.5	OpenAI	Generative pretrained transformer	Proprietary	Estimated 175 billion

^a^LLaMA: large language model Meta AI.

^b^LLM: large language model.

We chose FMs that are considered “small” LLMs with 7 billion parameters. If the CaLM can be developed using a “small” FM, it will make powerful AI capabilities more accessible, affordable, and versatile. The models can be deployed using regular computing infrastructure available in low-resource settings such as small community organizations and homes. The open-source FMs used in this project have permissive licensing models that can potentially be deployed in low-resource settings. We also included a “large” LLM (proprietary GPT-3.5 with an estimated 175 billion parameters owned by OpenAI [17,35,36]) as a point of comparison to highlight the potential that small FMs have for developing the CaLM.

### Caregiving Corpus and Knowledge Base

The knowledge base is central to the CaLM in providing and guiding the FM with a rich context of caregiving-specific information. The long-term goal of this study is to develop a CaLM for various care recipients’ conditions. In this preliminary work, we focused on developing a CaLM for caregivers of individuals with Alzheimer disease and related dementias (ADRD). The development of the caregiving knowledge base started with the development of the caregiving corpora. In this project, we developed a caregiving corpus related to ADRD by gathering a significant collection of data related to ADRD caregiving. These data included publicly available journal articles, care guidelines, and practical insights from online caregiver discussion forums. Table 2 describes the data sources that were collected for the knowledge base.

**Table 2:** Sources for the caregiving corpus.

Source	Source file format	Type	Document extracted	Number of documents (N=8568)	Number of chunks (N=196,926)
caregiver.com	HTML	Online caregiving forum, discussion, and tips	High-quality questions and answers	142	1591
agingcare.com	HTML	Caregiver support website	Caregiving resources and questions and answers	402	4169
alzconnected.org	HTML	Discussion forums from Alzheimer’s Association	Low-technical resources in question and answer format	4087	4087
deliriumnetwork.org	PDF	Repository of resources related to delirium	High-quality literature and resources	1714	72,426
PubMed	PDF	Database of journal articles	High-quality literature and resources	2195	114,383
nia.nih.gov	PDF + HTML	Education resources on aging and ADRD^a^	Literature and caregiving resources	9	133
alzheimers.gov	HTML	Education resources on aging and ADRD	Literature and caregiving resources	8	55
alzheimers.org.uk	HTML	Education resources on aging and ADRD	Literature and caregiving resources	7	39
alz.org	HTML	Education resources on aging and ADRD	Literature and caregiving resources	2	12
Other web sources	HTML	Web article	Caregiving resources	2	31

^a^ADRD: Alzheimer disease and related dementias.

We selected ADRD for the proof-of-concept development of the CaLM owing to the substantial and growing impact these conditions have on the global population, especially within the aging demographic [37,38]. The intricate care requirements associated with the cognitive and behavioral symptoms of ADRD present a complex challenge that caregivers must navigate, often without formal training [7,39]. Given the progressive nature of these conditions, caregivers are in need of long-term support and strategies, underlining the importance of a dedicated resource like the CaLM. By addressing ADRD, the model can provide substantial support to a vast community of caregivers who are frequently underserved when it comes to specialized care resources. Moreover, with an extensive amount of research and guidelines already available for ADRD, there is a rich foundation upon which to build a detailed and accurate knowledge base, making it a particularly suitable focus for the initial deployment of this innovative tool.

The documents we collected were crawled and downloaded from websites in various formats such as HTML, PDF, and plain text (Table 2). These raw documents then underwent several preprocessing stages before they were converted into a caregiving corpus. The preprocessing procedure included data format conversion, text cleaning, and document chunking. During data format conversion, documents in HTML and PDF were converted into either plain text markdown format or plain text. The converted documents were cleansed from errors of duplication and unnecessary characters, such as extra spaces, new lines, punctuations, and non-ASCII tags, during conversion using regular expression (Regex) rules.

The last stage of text processing was document chunking and converting the chunk documents into Document format with metadata. Document chunking broke down the documents in the caregiving corpus into smaller “chunks” with a length of 1200 characters each to fit the FM context window limitation and reduce unrelated text in the generated prompt in the RAG system. These chunks of text were then converted into a 768-dense dimensional vector using Siamese BERT-Network [40] as the embedding model. We used BAAI general embedding (BGE), a recently released embedding model that was pretrained on massive data sets and multiple tasks, as a general-purpose embedding model [41]. BGE was selected because it provides a good trade-off for memory usage, speed of computation, and embedding quality. These vectors were then stored in a Chroma DB vector database, and it functioned as the retriever database for the RAG system. Chroma DB was selected in this experiment for its ease of setup. It is not too strict on the data schematics and supports multiple built-in distance functions such as squared L2, inner product, and cosine similarity.

### Retriever Module in the CaLM: Providing Caregiving Context Prompts

The retriever module in the CaLM searches for semantically related information from the caregiving knowledge base to provide a caregiving context that matches users’ questions. The related caregiving information provides enhanced context that is appended to the user’s prompt and passed to the FM. It is important to implement a retrieval mechanism that can efficiently search through the caregiving knowledge base to respond to a user’s questions. A dense passage retriever (DPR) [42] is used to develop the retriever module in the CaLM. The DPR captures more complex semantic relationships between the query and the documents, leading to more accurate retrieval results.

In the retriever implementation, the user’s question is converted into a vector with the same embedding model used to encode the caregiving knowledge base. The DPR uses cosine similarity as the distance function to calculate related documents in the vector space by calculating the user’s question vector and existing document vectors inside the Chroma vector database. The most related documents were limited to 3 to keep the content relevant to the user’s question, avoid FM token window limitations, and prevent degrading model generation quality [43].

Implementing the CaLM using a RAG framework has 2 main benefits. It ensures that the model has access to the most current reliable facts about caregiving, and it provides users with access to the model’s sources, ensuring that its claims can be checked for accuracy and can ultimately be trusted. The caregiving knowledge base is designed to be updated regularly. The knowledge base in the CaLM can be updated regularly, and the model can be retrained more frequently. Updating the knowledge base and fine-tuning the FM require fewer resources than retraining FMs. The model had access to the most current and reliable facts because of the frequency of the updates and the fine-tuning.

### Fine-Tuning the FM in the CaLM

The CaLM uses a fine-tuned FM to synthesize an accurate answer tailored to the language and nuances of caregiving. The fine-tuned FM is an original FM that has been retrained using supervised learning on Q-A pairs related to caregiving. Fine-tuning involves retraining the FM on a caregiving domain-specific data set. Full fine-tuning that involves updating all of an FM’s parameters is less feasible because it requires large computing resources. A more practical and commonly used type of fine-tuning is called parameter-efficient fine-tuning (PEFT), which requires retraining only part of or an extra component of the pretrained FM. The most widely used PEFT technique is called LoRA (low-rank adaptation) [44], which adds a small number of trainable parameters to the FM while the original model parameters remain frozen. In developing the CaLM, we used LoRA as well as the quantized technique of LoRA called QLoRA [45] to improve the FM’s memory efficiency during retraining.

The main benefit of using RAG in the CaLM is that by grounding an FM on a set of external verifiable facts about caregiving, the model has fewer opportunities to pull information baked into its parameters, thus reducing the chances that the model will “hallucinate” incorrect or misleading information. This is critical in achieving the goal of a reliable CaLM.

### Data Sets for Fine-Tuning the FM and for Model Evaluation

Recent studies indicate that an FM can be made more accurate by fine-tuning it on a high-quality smaller data set [46-49]. In this study, we used a large data set for the RAG framework’s knowledge base and a high-quality small data set for fine-tuning the FM. The goal was to further improve the performance of the FM by retraining it using a high-quality data set tailored for caregiving Q-A so that it can respond more accurately and in a more contextually relevant way to questions related to caregiving. Fine-tuning is important particularly if we want to use a smaller and more efficient FM to achieve the goal of an accessible CaLM.

The high-quality training set to fine-tune the FM was in the form of Q-A pairs, which were developed using a combination of both automatic and manual methods. Fragments of documents in the caregiving knowledge used in the RAG approach were randomly sampled and selected, and they were used as seed context to generate questions and answers. OpenAI GPT-4 was used to generate variations of questions based on the seed contexts. This seed of questions was then paired with the 3 documents in the knowledge base that were most closely related to the questions to synthesize the output answer. The pairs of Q-A data sets were subsequently curated manually by a data annotator to validate that the questions were representative from a caregiver perspective and that the answers and their associated references were correct. The data annotator was responsible for selecting which Q-A pairs were relevant to caregiving. Following the curation, the data set was deduplicated to remove duplicate data, prevent data leakage, and improve the fine-tuning process [49].

We constructed 581 Q-A pairs of data sets, each of which included questions and answers with references. Of these 581 pairs, we randomly selected 415 for inclusion in a training subset, reserving the remaining 66 pairs as a test subset. The 415 Q-A pairs in the caregiving training set were used for fine-tuning the FMs using supervised learning. Multimedia Appendix 1 provides an example of the fine-tuning data set. The goal of the fine-tuning process is to adapt the pretrained FM to the caregiving field so that it can respond more accurately and with more contextual relevance to questions related to caregiving. Once all the components of the CaLM were developed and trained, the model was evaluated using the 66 pairs of questions that were not included in the training.

We evaluated the performances of the models using the test set consisting of 66 Q-A pairs. The evaluation was conducted after the components of the RAG framework were developed and the small FMs were fine-tuned by retraining the models on the training set of 415 Q-A pairs. The training set (comprising 415 entries) and the test set (comprising 66 entries) were distinct with no overlapping questions or content. After exposing the FMs to the questions from the test set, we checked the FMs’ answers against the test set answers to determine whether the FMs were outputting accurate answers to the test questions. The performance of the FMs was measured by the similarities between the reference answers to the test set questions and the output generated by the models. In addition to similarities, we evaluated the capabilities of the models in providing references to the answers.

We tested the small FMs trained in 3 different settings: vanilla, RAG, and RAG + fine-tuned. Vanilla is the original FM without adding the caregiving knowledge base. In the RAG setting, for every question in the data, additional context from the knowledge base was added before the Q-A pair was sent to the FM. In the RAG + fine-tuned setting, we followed the same procedure as with the RAG setting, except that the FM had already been retrained using the 415 Q-A training set pairs. We compared the small FMs with OpenAI GPT 3.5 as a baseline benchmark. Because GPT 3.5 is a proprietary commercial system, we were not able to control its fine-tuning variables other than providing examples. Therefore, we cannot compare it with other FMs that were retrained using the caregiving Q-A training set.

### Ethical Considerations

To demonstrate a proof-of-concept for the CaLM, we generated a caregiving corpus specifically focused on ADRD. This corpus was compiled from a comprehensive set of publicly available sources, including journal articles, caregiving guidelines, and content from online caregiver discussion forums such as posts and replies. All data sources used were already in the public domain and freely accessible to the general public. Therefore, this study did not fall under the purview of institutional review board (IRB) oversight, and it was deemed exempt from IRB review by the University of Pittsburgh.

Despite this exemption, we adhered to strict ethical guidelines, including the deidentification of any potentially identifiable information extracted from online sources. This precaution ensures that individual privacy is maintained and aligns with ethical research standards, particularly those concerning the use of social media data in research.

## Results

### Experiment Results

We applied the benchmark metrics commonly used in evaluating language models, including BLEU (Bilingual Evaluation Understudy), ROUGE (Recall-Oriented Understudy for Gisting Evaluation), CHR-F (character N-gram F-score), and BERT (Bidirectional Encoder Representations from Transformers) score. These metrics are automatic evaluations that measure the similarity of a response to a provided reference answer in the test set. The metrics are considered to have a high correlation with human judgments of quality. BLEU is the oldest and most popular metric, and it captures word-level similarities between the answer and the reference. ROUGE evaluates how well the models produce a generated answer compared to the reference answer by measuring several overlapping units such as n-gram, word sequences, and word pairs [50]. The scores for the BLEU and ROUGE metrics range between 0 and 1, with 1 being a perfect score. CHR-F measures similarities at the character level, and it is scored between 0 and 100, with 100 being a perfect score. The BERT score calculates the similarity between the output and a reference using sentence representation and focuses on measuring semantic similarity. The BERT metric is scored between 0 and 1, with 1 being a perfect score.

**Figure 2:**
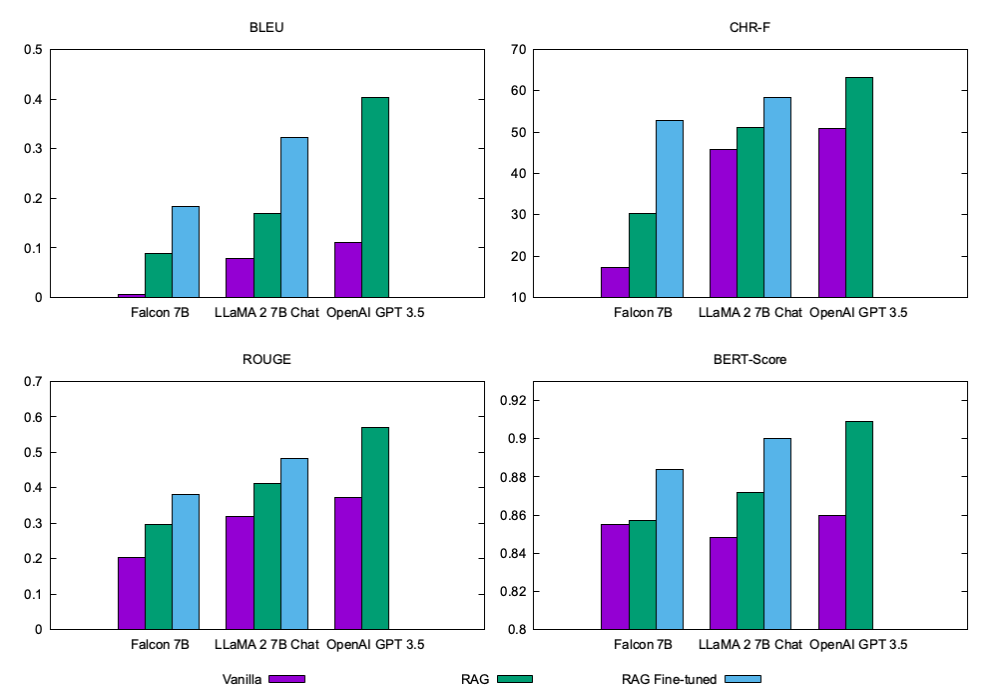
Benchmarks of the Falcon 7B, LLaMA 2 7B Chat, and OpenAI GPT 3.5 models in 3 different approaches: vanilla, RAG, and RAG + fine-tuned (except GPT 3.5). BERT: Bidirectional Encoder Representations from Transformers; BLEU: Bilingual Evaluation Understudy; CHR-F: character N-gram F-score; LLaMA: large language model Meta AI; RAG: retrieval augmented generation; ROUGE: Recall-Oriented Understudy for Gisting Evaluation.

The purpose of using multiple metrics was to provide a more comprehensive intrinsic automatic evaluation and to assess whether the performances are consistent across different standards of granularity, from character to word and meaning. The performance is summarized in Figure 2. In addition to the general performance of the language models for providing the right answers, we evaluated their reliability by measuring their capacity to provide references with the answers. The capability of the models in providing references is summarized in Table 3.

The RAG framework improved the performance of all FMs used in this study across all measures. The performances improved significantly at the character (CHR-F), word (BLEU and ROUGE), and semantic (BERT score) levels. As expected, the large FM (OpenAI GPT 3.5 with estimated 175 billion parameters) performed better than the small FMs (Falcon 7B and LLaMA 2 7B with 7 billion parameters) across all metrics. The larger FM has more parameters and is able to accommodate having more knowledge encoded into it.

The RAG framework that was implemented with fine-tuning performed better than RAG-only and vanilla for the 2 small FMs. Because we could not retrain the GPT 3.5 model, we were not able to evaluate a retrained (fine-tuned) version of it. The most interesting result is that RAG + fine-tuned LLaMA 2 7B performed significantly better than vanilla GPT 3.5 across all metrics. Fine-tuned Falcon 7B also performed better than vanilla GPT 3.5 across all metrics. LLaMA 2 7B and Falcon 7B have only 7 billion parameters, while OpenAI GPT 3.5 has 175 billion parameters. This shows that a small FM with the injection of domain-specific knowledge can perform better than a much larger FM.

**Table 3:** Capability to return references in the answer.

Foundation model and variant			Returning references, n		Not returning references, n		Returning references, %	Correct references (human evaluation), %			
**Falcon 7B**											
	RAG^a^	0		66		0		0			
	RAG + fine-tuned	61		5		92		66			
**LLaMA^b^ 2 7B - Chat **											
	RAG	17		49		26		12			
	RAG + fine-tuned	66		0		100		80			
**GPT 3.5**											
	RAG GPT 3.5	46		20		70		62			

^a^RAG: retrieval augmented generation.

^b^LLaMA: large language model Meta AI.

In addition to the general performance of the language models in providing the right answers, we also evaluated their reliability by measuring their capacity to provide accurate references with the answers. Table 3 provides results on the capability of the models to return references in their answers and mentions the number of correct references. The references provided by the models were evaluated for correctness and relevance. In addition to checking whether the generated answer returned a list of references, the annotator verified that the links were correct and active. The annotator also checked each inline reference to determine if the content in the answer was part of the original document. Using the results from these checks, the data annotator decided whether each reference was correct and relevant to the answer.

None of the vanilla FMs were trained to return references, and therefore, no references were provided for any questions (the results were all zeroes). LLaMA 2 7B in the RAG + fine-tuned setting provided references to all 66 answers. It performed better than GPT 3.5 with the RAG framework, which returned references in only 46 of the 66 answers (70%). Fine-tuning by retraining the FMs using the 415 Q-A pairs of the training set significantly improved the capabilities of the FMs to return references. The percentage of correct and relevant references among the models followed similar patterns, with LLaMA 2 7B performing better than GPT 3.5 (80.0% vs. 62.1%). Multimedia Appendix 2 provides examples of comparisons between the generated answers provided by the CaLM and GPT 3.5, as well as reference answers from the Q-A test set.

Implementing the RAG framework in the development of the CaLM had several benefits. First, we were able to ensure that the language model had access to the most current facts. The caregiving knowledge could be updated regularly, and we could retrain the model more frequently than we would have been able to when using a new FM. Updating the knowledge base and fine-tuning FMs require fewer resources than retraining FMs. Second, the model had access to the most reliable facts because of the frequency of the updates and the fine-tuning that included references in the knowledge base and the training set. Third, users had access to the model’s information sources, ensuring that its answers could be checked for accuracy and ultimately trusted.

### Developing a Caregiver Chatbot Using the CaLM

**Figure 3:**
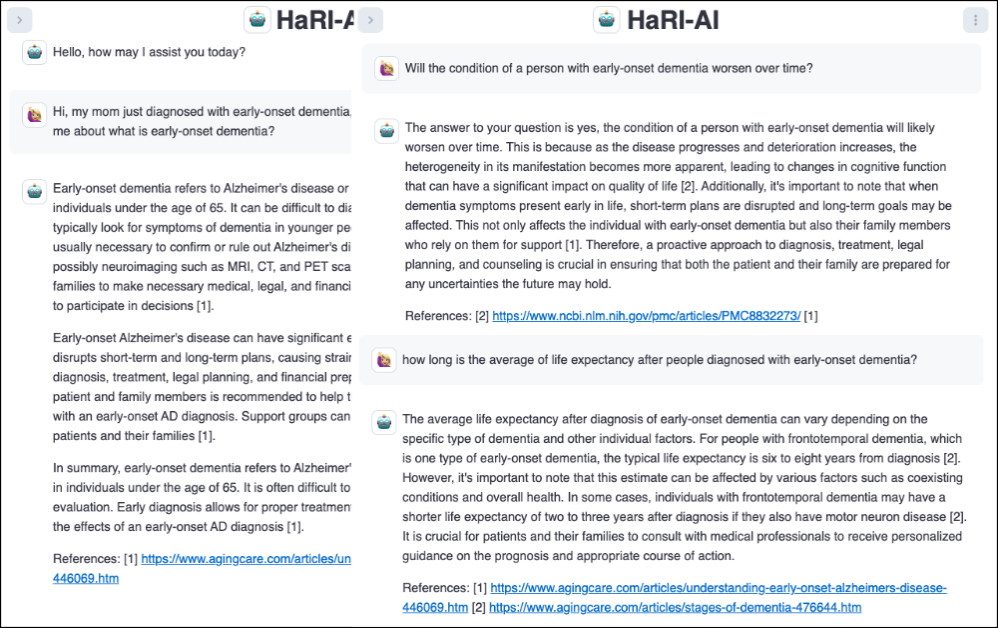
Conversation example of a caregiver chatbot that uses the caregiving language model.

We developed a prototype of a caregiver chatbot that uses the CaLM as the engine. The purpose of the chatbot prototype was to show that the CaLM is accessible and can be deployed in a low-resource environment. The chatbot interface and example of the interaction are illustrated in Figure 3. We developed the user interface and interaction system that work with the CaLM engine. The model that powers the chatbot is small and requires approximately 4-6 GB of memory. The chatbot can be deployed on a laptop or a small server.

## Discussion

### Principal Findings

The findings of this study showed that a domain-specific language model for caregiving can be developed by using the RAG framework. The RAG framework improved the quality of answers generated by FMs in the domain-specific caregiving field by grounding the model on a caregiving knowledge base to supplement the general knowledge already stored in the internal representation of the FM. The results were consistent across different metrics, showing that FMs that were adapted using the RAG framework enriched with a caregiving knowledge base performed better than the original vanilla FMs. Performance was further improved when the FMs were fine-tuned by retraining them using supervised learning on a specific Q-A training set related to caregiving. Fine-tuning also improved the reliability of the models by increasing their capability to provide verifiable responses through references. The results showed that a reliable CaLM can be developed by combining FMs with the RAG framework and by fine-tuning involving retraining the FMs.

The study found that small FMs can perform comparably to or better than much larger FMs when they are grounded in domain-specific knowledge related to caregiving. For example, LLaMA 2 7B (7 billion parameters) with access to a caregiver knowledge base performed better than GPT 3.5 with 175 billion parameters. Fine-tuned LLaMA 2 7B also provided reliable answers by supplying references to all of the answers in the test set. Smaller FMs require less computing power to train and fewer resources to deploy once they are trained. For example, LLaMA 7B with RAG and fine-tuning can be deployed using the computing power of small servers or desktop computers. This makes it more accessible for small organizations that want to develop language models specific to the domains important to them. Other methods for developing “small” FMs that can perform almost as well as large FMs have been proposed in recent works, and an example is the Starling-7B LLM [51], which uses reinforcement learning from AI feedback (RLAIF) and high-quality large training data to enhance model performance.

The implications of the results show the potential for developing reliable and accessible language models in domain-specific areas by combining smaller fine-tuned FMs with the RAG framework, as evidenced by other studies [52,53]. The parameter sizes available for FMs will continue to grow rapidly in the coming years. For example, the latest release of LLaMA 2 is available in 3 model sizes: 7, 13, and 70 billion parameters. The computing power accessible to smaller organizations will also continue to grow. Therefore, this approach will still have potential even when the sizes of FMs increase. By using an FM size that can be supported by the infrastructure available to small organizations, we can ensure that the CaLM will remain accessible to those organizations. For example, FMs with a size of 70 billion will likely be accessible within the next few years. Using smaller FMs with RAG + fine-tuning will remain a valid approach even as large FMs grow even larger because hallucinations will still exist in the large FMs.

Two main methods were used in the development of the CaLM: RAG and fine-tuning of FMs. Fine-tuning has a number of drawbacks, including the computational resources needed for full parameter fine-tuning and the risk of catastrophic forgetting, where the model loses its ability to perform well on the original task or domain after being fine-tuned with new data [54]. A previous study found that the PEFT technique is effective in preventing the phenomenon known as “catastrophic forgetting” in original FM capabilities compared with the full-parameter fine-tuning technique [55]. Therefore, fine-tuning with PEFT will increase model capability on the CaLM’s downstream tasks without sacrificing original FM capabilities.

The CaLM is reliable and accessible and has significant potential for solving downstream tasks through the development of systems such as a chatbot for caregiving. This approach can also be used for developing systems for caregivers that are specific to the conditions of the care recipients they serve, such as for caregivers of individuals with disabilities or cancer. The contextual reference in the caregiving knowledge base can be further tailored to organizational needs or services. For example, if an organization has a service related to long-term care, the language model can be tailored using the organization’s internal documents, procedures, and guidelines. We can also tailor the answer function to the desired communication styles or education levels of the intended users.

### Limitations

Although the study shows the potential of the development of a reliable and accessible language model in a specific domain, such as caregiving, it has a number of limitations. The first limitation is that the knowledge base is restricted to the caregiving of individuals with ADRD. The choice to focus primarily on ADRD was driven by the significant global impact of this condition and the availability of extensive research and guidelines. However, this focus may limit the broader applicability of our findings to other caregiving contexts, potentially affecting the model’s generalizability. To address this, future work will include expansion of the caregiving corpora related to other care recipient conditions, especially chronic and complex conditions. The corpora related to issues and skills in general caregiving (irrespective of the care recipient’s condition) used in this study are also limited. We plan to expand the caregiving corpora by retrieving all publicly available documents related to caregiving. We envision the CaLM as an iterative and evolving model. By integrating data and insights from broader caregiving contexts, we aim to evolve the CaLM into a more inclusive and representative model, catering to the diverse needs and challenges encountered in caregiving.

The study used quantitative metrics for evaluating the performance of the language models. Quantitative metrics show consistency across different metrics. The benefits of quantitative metrics include reduced time and cost and increased consistency compared to human evaluation. All metrics used in this study fall into the category of intrinsic metrics [56]. Intrinsic metrics measure the proficiency of an LLM in generating coherent and meaningful sentences relying on language rules and patterns [57]. However, these quantitative metrics are insufficient for capturing the multifaceted human perspectives and the practicalities encountered in real-world caregiving scenarios. Future research should include evaluations using extrinsic metrics that are crafted to encapsulate user experiences and the actual applicability of language models within real-world settings [57]. Extrinsic metrics that are relevant to the health care and caregiving domains should measure the accuracy and reliability of information, its timeliness and relevance, and the system’s capability to provide empathetic and emotionally supportive responses [56]. The next phase of research will aim to incorporate these metrics and engage real family caregivers and health care professionals to evaluate the quality of the answers. Because the CaLM will be implemented as a chatbot that engages family caregivers, assessments using psychological dimensions, such as perceived humanness, likeability, anthropomorphism, animacy, and perceived safety [58], need to be included in more holistic evaluations in the future.

Additionally, ethical considerations are integral to the deployment of AI in caregiving. The AI system must employ stringent data privacy measures and transparency in its decision-making processes [59-61]. This transparency ensures that caregivers can trust and understand the rationale behind AI-generated advice and recommendations. Moreover, addressing potential biases in the training data is crucial to ensure that the AI system provides equitable support across all user demographics. Future development of the CaLM will involve continuous engagement with stakeholders to address these ethical challenges and ensure the model’s alignment with the highest standards of responsible AI practice.

### Conclusions

This study shows promise in the development of CaLMs. It shows that the CaLM developed using the RAG framework and FM fine-tuning can provide reliable answers to user questions by retrieving accurate references. The study shows that a reliable CaLM can be developed using FMs with a knowledge base specific to the caregiving domain. It also shows that a small FM that uses a caregiving knowledge base and is retrained using caregiving Q-A sets can perform better and more reliably than a much larger FM in answering caregiving-related questions. The CaLM developed using small FMs performed better than the benchmark large FM (OpenAI GPT 3.5), which will allow the CaLM to be accessible and deployed in low-resource settings. Future work includes expanding the domain knowledge to include other conditions of care recipients to enhance utility. Furthermore, the evaluation process will be refined by engaging caregivers as end users in providing feedback, alongside insights from health care professionals and caregiving domain experts.

## Appendix

Multimedia Appendix 1 Examples of questions and answers for training (fine-tuning) foundation models.

Multimedia Appendix 2 Examples of answers generated by the caregiving language model (CaLM) and GPT 3.5.

## Abbreviations

ADRD: Alzheimer disease and related dementias
AI: artificial intelligence
BERT: Bidirectional Encoder Representations from Transformers
BGE: BAAI general embedding
BLEU: Bilingual Evaluation Understudy
CaLM: caregiving language model
CHR-F: character N-gram F-score
DPR: dense passage retriever
FM: foundation model
LLaMA: large language model Meta AI
LLM: large language model
LoRA: low-rank adaptation
PEFT: parameter-efficient fine-tuning
Q-A: question and answer
RAG: retrieval augmented generation
ROUGE: Recall-Oriented Understudy for Gisting Evaluation

## Data Availability

The data sets generated and analyzed during this study, including the caregiving corpus and knowledge base, are available from the corresponding author upon reasonable request. The prototype caregiving language model developed in this study, which focuses on Alzheimer disease and related dementias, is not publicly accessible at this time. This is due to ongoing improvements and optimizations being made to enhance its performance and reliability. Interested individuals who wish to access the model or the data sets for research purposes are encouraged to contact the corresponding author.
