# Supplementary material for: A Reliable and Accessible Caregiving Language Model (CaLM) to Support Tools for Caregivers: Development and Evaluation Study
Source: JMIR Form Res. 2024 Jul 31;8:e54633. doi: 10.2196/54633 (PMC11325100; doi:10.2196/54633)
Supplement: Multimedia Appendix 1 [file formative_v8i1e54633_app1.docx]

**Multimedia Appendix 1.** Examples of questions and answers for training (fine-tuning) foundation models.

| System Prompt | Question | Context | Answer |
| --- | --- | --- | --- |
| Generate a comprehensive, informative and helpful answer for given question solely based on the information provided (Source and Content). You must only use information from the provided contents. Combine content information together into a coherent answer. Do not repeat text. Anytime you cite piece of information for the answer, follow by add its numerical source in ```[]``` format after text you cite. Only cite the most relevant content that answer the question accurately. Format list of References in this markdown format ```[index](Source link)``` and added at the end. If a question does not make any sense, or is not factually coherent, explain why instead of answering something not correct. If provided information is not providing enough related information to answer the question don't make up an answer. If you don't know the answer to a question, say you don't have answer and please don't share false information. | What communication strategies can I use to help my loved one with dementia understand and express their needs? | Content: How to communicate with a person with dementia? We aren’t born knowing how to communicate with a person with dementia—but we can learn. Improving your communication skills will help make caregiving less stressful and will likely improve the quality of your relationship with your loved one. Good communication skills will also enhance your ability to handle the difficult behavior you may encounter as you care for a person with a dementing illness. Set a positive mood for interaction. Your attitude and body language communicate your feelings and thoughts more strongly than your words do. Set a positive mood by speaking to your loved one in a pleasant and respectful manner. Use facial expressions, tone of voice, and physical touch to help convey your message and show your feelings of affection. Get the person’s attention. Limit distractions and noise—turn off the radio or TV, close the curtains or shut the door, or move to quieter surroundings. Before speaking, make sure you have her attention; address her by name, identify yourself by name and relation, and use nonverbal cues and touch to help keep her focused. If she is seated, get down to her level and maintain eye contact.Source: [1] <https://www.caregiver.org/resource/caregivers-guide-understanding-dementia-behaviors/>Content: What are some tips to communicate with a dementia patient? We aren’t born knowing how to communicate with a person with dementia—but we can learn. Improving your communication skills will help make caregiving less stressful and will likely improve the quality of your relationship with your loved one. Good communication skills will also enhance your ability to handle the difficult behavior you may encounter as you care for a person with a dementing illness. Set a positive mood for interaction. Your attitude and body language communicate your feelings and thoughts more strongly than your words do. Set a positive mood by speaking to your loved one in a pleasant and respectful manner. Use facial expressions, tone of voice, and physical touch to help convey your message and show your feelings of affection.Source: [2] <https://www.caregiver.org/resource/ten-tips-for-communicating-with-a-person-with-dementia/>Content: or finding a local in-person caregiver support group. Doing so will put you in touch with other caregivers who can share their own experiences, listen to your frustrations, and offer their sage advice and answers. Improve communication Conversations with someone who has dementia can often feel challenging. Improve communication with your loved one by following these tips for talking with dementia patients : Limit distractions Speak clearly and simply; using hand gestures can help Maintain eye contact Talk about one thing at a time Be patient, and understand that some days may be better or worse than others Manage sleep and behaviors With dementia comes a myriad of behavioral changes, like sleep issues, wandering, confusion, and angry outbursts. These can all present major challenges for patients and caregivers alike. To help manage a loved one’s problematic behaviors, try the following tactics: To minimize wandering, make sure all doors in the house are secured, and consider installing a surveillance system or alarms to help keep your loved one safe. For sleep issuesSource: [3] <https://www.agingcare.com/articles/alzheimers-presents-unique-challenges-for-home-care-154829.htm> | When communicating with a person with dementia, there are several strategies that can help them understand and express their needs. First, it is important to set a positive mood for interaction. This can be done by speaking to your loved one in a pleasant and respectful manner, using facial expressions, tone of voice, and physical touch to convey your message and show your feelings of affection [1][2]. Getting the person's attention is also crucial. Limit distractions and noise, such as turning off the radio or TV, closing the curtains or shutting the door, or moving to quieter surroundings. Before speaking, make sure you have their attention by addressing them by name, identifying yourself by name and relation, and using nonverbal cues and touch to help keep them focused. If they are seated, get down to their level and maintain eye contact [1]. When engaging in conversation, it is important to speak clearly and simply. Using hand gestures can also be helpful in conveying your message. It is best to talk about one thing at a time and be patient, understanding that some days may be better or worse than others [3]. In addition to communication strategies, it is important to manage sleep and behaviors in individuals with dementia. Sleep issues, wandering, confusion, and angry outbursts are common behavioral changes associated with dementia. To help manage these behaviors, it is recommended to secure all doors in the house to minimize wandering. Installing a surveillance system or alarms can also help keep your loved one safe. For sleep issues, it may be helpful to establish a consistent bedtime routine and create a calm and comfortable sleep environment [3]. Overall, improving communication skills and implementing strategies to manage behaviors can greatly enhance the caregiving experience and improve the quality of the relationship with your loved one with dementia. References:[1] https://www.caregiver.org/resource/caregivers-guide-understanding-dementia-behaviors/[2] https://www.caregiver.org/resource/ten-tips-for-communicating-with-a-person-with-dementia/[3] https://www.agingcare.com/articles/alzheimers-presents-unique-challenges-for-home-care-154829.htm |
