# Supplementary material for: A Reliable and Accessible Caregiving Language Model (CaLM) to Support Tools for Caregivers: Development and Evaluation Study
Source: JMIR Form Res. 2024 Jul 31;8:e54633. doi: 10.2196/54633 (PMC11325100; doi:10.2196/54633)
Supplement: Multimedia Appendix 2 [file formative_v8i1e54633_app2.docx]

**Multimedia Appendix 2.** Examples of answers generated by the caregiving language model (CaLM) and GPT 3.5.

| **No** | **Question** | **Reference Answer** | **CaLM Answer (RAG + Fine-Tuned Model)** | **GPT3.5 Answer** |
| --- | --- | --- | --- | --- |
| 1 | What are the benefits of hospice care for individuals in the advanced stages of Alzheimer's disease? | Hospice care offers several benefits for individuals in the advanced stages of Alzheimer's disease. Firstly, hospice services are designed to support individuals at the end of life, providing specialized care and support during this challenging time. Hospice services may include support groups, visiting nurses, pain management, and home care. These services aim to improve the quality of life for the patient, ensuring their comfort and well-being.  In the late stage of Alzheimer's disease, individuals can be frail and susceptible to infections. Hospice care focuses on managing pain and other symptoms, helping the patient remain comfortable. This is particularly important as the side effects of many drug therapies can outweigh the benefits in this stage. Hospice teams are experienced in providing symptom management and can offer helpful suggestions to family and friends on how to connect with their loved one during the late stages of dementia.  Additionally, hospice care benefits families by offering caregiving assistance and emotional support. Caring for a loved one with advanced Alzheimer's disease can be overwhelming, and hospice services can provide much-needed support to the entire family. Hospice teams can help alleviate the burden of caregiving and provide emotional guidance during this difficult time.  In summary, hospice care provides specialized support and services for individuals in the advanced stages of Alzheimer's disease. It focuses on improving the patient's quality of life, managing symptoms, and providing assistance and emotional support to the family. | Hospice care can provide numerous benefits for individuals in the advanced stages of Alzheimer's disease, including support and comfort during the end-of-life process. It is important to note that hospice services are usually arranged through the treating physician and are typically not available until it is anticipated that a person has less than six months to live [1].  In terms of benefits for individuals in advanced stages of Alzheimer's disease, hospice care can help manage pain and other symptoms, providing relief from discomfort. Additionally, hospice teams can offer helpful suggestions on how family members and friends can continue to connect with their loved ones during the late stages of dementia [2].  Hospice services also provide support for families dealing with end-of-life care challenges. This includes offering emotional support and assistance in caring for individuals at home or in a skilled nursing facility/dementia care facility, where adequate management and supervision can be provided [1].  In summary, hospice care offers benefits such as pain management, symptom control, connection opportunities with loved ones during the late stages of dementia, emotional support for families, and assistance in caring for individuals at home or in a skilled nursing facility/dementia care facility. These advantages can greatly improve the quality of life for individuals in advanced stages of Alzheimer's disease.  References:  [1] https://www.caregiver.org/resource/alzheimers-disease-caregiving/  [2] https://www.agingcare.com/articles/can-dementia-be-fatal-476368.htm | Hospice care can provide numerous benefits for individuals in the advanced stages of Alzheimer's disease. Some of the key benefits include:  1. Comfort and pain management: Hospice care focuses on making the individual as comfortable as possible, managing their pain and ensuring their quality of life. This is particularly important for individuals with Alzheimer's disease who may experience discomfort or pain due to physical symptoms or complications.  2. Emotional and psychological support: Hospice care teams include professionals such as social workers, counselors, and chaplains who provide emotional and psychological support not just to the patient but also to their family members. They help individuals cope with the challenges and emotional stress that come with Alzheimer's disease.  3. Customized care plans: Hospice care teams work closely with the patient, family, and medical professionals to develop a personalized care plan that meets the specific needs and preferences of the individual. They consider factors such as the stage of Alzheimer's disease, cognitive abilities, and any other existing medical conditions.  4. Enhanced quality of life: By providing specialized care, hospice teams aim to improve the quality of life for individuals in the advanced stages of Alzheimer's disease. They focus on maintaining dignity, maximizing comfort, and promoting a sense of well-being for the patient.  5. Assistance with daily tasks: As Alzheimer's disease progresses, individuals may struggle with performing essential activities of daily living (ADLs) such as eating, bathing, and dressing. Hospice care can provide assistance with these tasks, helping individuals maintain their independence and dignity for as long as possible.  6. Family education and guidance: Hospice care teams educate and guide family members on how to best care for their loved ones with Alzheimer's disease. They provide information on symptom management, communication techniques, and strategies for providing comfort and support.  7. Bereavement support: When the individual with Alzheimer's disease eventually passes away, hospice care teams continue to provide support to the family during the grieving process. They offer counseling, support groups, and resources to help family members navigate their loss and find healing.  It's important to note that hospice care is typically provided in the individual's home or in a specialized hospice facility, allowing them to receive care in a familiar and comfortable environment. The goal of hospice care is to improve the overall well-being of individuals with advanced Alzheimer's disease and support their families throughout the progression of the disease. |
| 2 | What are the early signs of dementia? | The early signs of dementia can vary from person to person, but there are common warning signs to watch for. Memory loss is a key sign of dementia, where individuals may repeat themselves or rely on aids such as sticky notes to remember things. Forgetting the names of familiar friends and family members is also a sign of dementia. It's important to note that memory loss from dementia is not a normal part of aging, and forgetting entire events due to short-term memory loss is cause for concern. | The early signs of dementia can vary depending on the cause, but there are some common symptoms to watch for. Alzheimer's disease is the most common type of dementia and its warning signs may be familiar. However, symptoms can go unnoticed for a while even if you are caring for someone with Alzheimer's [1].  Some early signs of Alzheimer's include wandering and getting lost, repeating questions or sentences, difficulty recognizing friends and family, impulsive behavior, and experiencing hallucinations or delusions. It is important to note that these symptoms may be similar to other types of dementia [1].  In addition to Alzheimer's disease, there are other types of dementia with distinct early warning signs. Lewy body dementia has some symptoms similar to Alzheimer's but also includes experiencing hallucinations or delusions, disorganized or illogical ideas, muscle rigidity, and reduced facial expression [1].  Frontotemporal dementia is another type of dementia with different early warning signs. It may include experiencing hallucinations or delusions, disorganized or illogical ideas, muscle rigidity, and insomnia [1].  In summary, the early signs of dementia can vary depending on the cause but common symptoms to watch for include memory loss, repeating questions or sentences, difficulty recognizing friends and family, impulsive behavior, experiencing hallucinations or delusions, disorganized ideas, muscle rigidity, reduced facial expression, and insomnia. If you notice any of these signs in your loved one, it is important to consult with a doctor who can run tests to determine the cause [1].  References:  [1] https://www.agingcare.com/articles/alzheimers-disease-dementia-warning-signs-144253.htm | Dementia is a progressive neurological disorder that affects cognitive function, memory, and behavior. While the symptoms of dementia may vary depending on the type and stage of the disease, there are some early warning signs that may indicate the presence of dementia. These signs can include:  1. Memory loss: Difficulty remembering recent events or conversations, forgetting important dates or appointments, and relying on memory aids or family members for reminders.  2. Difficulty with language and communication: Struggling to find the right words, repeating phrases or stories, or having trouble following and participating in conversations.  3. Confusion and disorientation: Becoming disoriented in familiar surroundings, getting lost on familiar routes, or having trouble understanding time, date, or location.  4. Impaired judgment and decision-making: Exhibiting poor judgment or making uncharacteristic decisions, such as giving away money or falling for scams.  5. Decline in problem-solving abilities: Having difficulty with tasks that require planning, problem-solving, and critical thinking skills, such as managing finances or following a recipe.  6. Personality and mood changes: Experiencing changes in mood, behavior, or personality, such as becoming more agitated, irritable, anxious, or apathetic.  7. Withdrawal from activities and social interactions: Losing interest in hobbies or social activities, avoiding social interactions, or becoming less engaged in previously enjoyed activities.  It's important to note that these signs can also be caused by other factors and may not necessarily indicate dementia. If you or someone you know is experiencing these symptoms, it is recommended to consult with a healthcare professional for a proper evaluation and diagnosis. |
| 3 | How can I ensure my loved one's safety at home, especially if they have a tendency to wander or become agitated | If your loved one has a tendency to wander or become agitated, there are several steps you can take to ensure their safety at home. First, it is important to reassure the person and speak to them calmly, listening to their concerns and frustrations. Allowing the person to maintain as much control in their life as possible is also important. Respecting their personal space and providing quiet times throughout the day, along with engaging activities, can help create a sense of security. Keeping well-loved objects and photographs around the house can also help the person feel more secure.  In terms of addressing wandering behavior, it may be helpful to identify triggers in the person's environment and make changes accordingly. For example, if the person tends to wander more in the evening hours, it could be attributed to activities they used to be responsible for during that time of day, such as getting the kids from school or fixing dinner. Redirecting the person with another activity before they start wandering can be effective. Engaging them in activities that provide a sense of purpose and accomplishment, such as folding laundry or sorting coins, can help alleviate restlessness. However, it is important to monitor all activities to ensure safety.  By implementing these strategies, you can help ensure the safety of your loved one at home, minimizing the risk of wandering and agitation. | To ensure the safety of your loved one at home, particularly when they tend to wander or become agitated, there are several steps you can take.  1. Reassure and speak calmly: Speak in a soothing voice and address their concerns and frustrations. Listen actively without interrupting them [2].  2. Allow control over personal space: Respect the person's autonomy by allowing them to maintain their personal space as much as possible [1].  3. Establish quiet times and engaging activities: Incorporate periods of calmness throughout the day, along with stimulating activities that they enjoy. This can help prevent restlessness and wandering [2].  4. Maintain familiar objects and photographs: Keeping well-loved items around the house provides a sense of security for your loved one [1].  5. Remind without prompting memory loss: If necessary, gently remind them who you are without pressing their memory too much. Avoid making assumptions about what they may or may not remember [2].  6. Encourage two-way conversations: Engage in active listening and allow for a back-and-forth conversation as long as possible. This can help maintain their sense of connection with you [1].  7. Distract through familiar activities: If communication becomes challenging, try distracting them with an activity they enjoy or find comfort in, such as reading a book or looking at photographs [2].  By implementing these strategies, you can create a safer environment for your loved one at home while also promoting their emotional well-being. However, if wandering becomes a significant concern and cannot be managed with the above measures, it may be necessary to explore additional safety solutions such as installing locks or alarms on doors that they tend to wander towards [2].  References:  [1] Tips for Caregivers and Families of People With Dementia (alzheimers.gov)  [2] https://www.agingcare.com/articles/wandering-top-tips-how-to-minimize-agitation-and-restlessness-432424.htm | Ensuring the safety of a loved one who has a tendency to wander or become agitated can be challenging, but there are several steps you can take to create a safer environment for them:  1. Secure the home: Install secure locks on doors and windows to prevent your loved one from leaving without your knowledge. Consider using alarms or chimes that will alert you if a door or window is opened. You may also want to consider adding deadbolts to exterior doors.  2. Remove hazards: Identify and remove any potential hazards in the home that could cause harm to your loved one. This may include removing loose rugs or furniture, securing electrical cords, and keeping the floors clear of clutter.  3. Utilize technology: Consider using technology to aid in their safety. For instance, you could install a video monitoring system to keep an eye on them remotely. There are also wearable devices available that can track their location or send alerts if they wander.  4. Establish a routine: Creating a predictable routine can help reduce agitation and confusion. Stick to a schedule for meals, medication, and activities. Having a consistent routine can provide a sense of comfort and stability.  5. Provide visual cues: Use visual cues throughout the home to help orient your loved one and provide reminders of important information. For example, you can place signs on doors to indicate the bathroom or bedroom, or use labels on drawers and cabinets to assist with finding items.  6. Ensure proper lighting: Good lighting is crucial to prevent falls and disorientation. Make sure all areas of the home are well lit, especially hallways, staircases, and entryways. Consider using night lights in the bedroom and bathroom to reduce confusion during nighttime.  7. Consider professional help: If your loved one's wandering or agitation becomes a constant concern, it might be beneficial to seek professional help. Consult with their healthcare provider or a geriatric specialist who can provide guidance and recommend appropriate interventions.  Remember, each situation is unique, and it's essential to tailor the safety measures to your loved one's specific needs. Regularly reassess their safety needs and make adjustments accordingly. |
| 4 | How do I obtain legal guardianship or power of attorney for a loved one with Alzheimer's? | To obtain legal guardianship or power of attorney for a loved one with Alzheimer's, it is crucial to make legal preparations for who will handle their medical and financial decisions on their behalf. Two important legal documents to consider are a durable power of attorney (POA) for health care and a durable power of attorney for finances. These documents appoint a trusted individual to oversee these areas of the patient's life once they are incapacitated.  To obtain these legal documents, it is recommended to consult with an elder law attorney who can assist in drawing up the POA documents and other essential estate planning documents such as wills, advance directives, and trusts. An elder law attorney specializes in legal matters related to the elderly and can provide guidance and expertise in navigating the legal process.  It is important to make these legal preparations as soon as possible because once a dementia patient is declared legally incompetent, they are unable to do so themselves. Taking proactive steps to establish legal guardianship or power of attorney can help ensure that the necessary decisions and arrangements are made in the best interest of the loved one with Alzheimer's.  In addition to legal preparations, it is also important to encourage Alzheimer's patients to stay mentally and physically active, as these factors may help slow memory loss and delay declines in functional abilities. Engaging in activities that the patient enjoys on a regular basis can be beneficial. Adopting a healthy diet is also recommended for overall well-being. | To obtain legal guardianship or power of attorney for a loved one with Alzheimer's, you can follow these steps:  1. Consult an Elder Law Attorney: It is recommended to consult an elder law attorney who specializes in estate planning and dementia-related matters [1]. They can guide you through the legal process of obtaining power of attorney for health care or finances, which are critical documents that appoint a trusted individual to handle medical and financial decisions on behalf of your loved one once they become incapacitated.  2. Determine Which Type of Power of Attorney: There are two types of durable powers of attorney commonly used for individuals with Alzheimer's disease - power of attorney (POA) for health care and POA for finances [1]. The first type focuses on making medical decisions, while the second pertains to financial matters. It is important to determine which one you need based on your loved one's specific situation.  3. Gather Required Documentation: Depending on the state or jurisdiction where your loved one resides, there may be specific requirements for obtaining power of attorney [1]. Your elder law attorney can inform you about these particular needs and help ensure that all necessary documents are prepared correctly.  4. Appoint a Trustworthy Individual: Once you have obtained the appropriate type of power of attorney, it is crucial to appoint an individual who is trustworthy and capable of handling your loved one's medical or financial matters [1]. This person should be someone who has their best interests at heart and can make informed decisions on their behalf.  5. Consider Estate Planning Essentials: In addition to power of attorney documents, it may be beneficial to consult with an elder law attorney regarding other estate planning essentials such as wills, advance directives, and trusts [1]. These legal elements can help ensure that your loved one's wishes are respected and their assets are managed properly.  6. Act Urgently: It is important to note that once a dementia patient is declared legally incompetent, they cannot make decisions regarding medical or financial matters themselves [1]. Therefore, it is crucial to act urgently when seeking legal guardianship or power of attorney for your loved one with Alzheimer's disease.  By following these steps and consulting an experienced elder law attorney, you can obtain the necessary legal documents that will allow someone trustworthy to handle medical and financial decisions on behalf of your loved one with Alzheimer's disease.  References:  [1] https://www.agingcare.com/articles/what-happens-after-alzheimers-diagnosis-154289.htm | Obtaining legal guardianship or power of attorney for a loved one with Alzheimer's can be a complex process, but it is essential to ensure their well-being and protect their interests. Here are the general steps to take:  1. Research local laws: Familiarize yourself with the laws and procedures regarding guardianship and power of attorney in the jurisdiction where your loved one resides. Laws can vary, so it's important to understand the specific requirements and processes.  2. Consult an attorney: Seek legal advice from an attorney experienced in elder law or estate planning. They can guide you through the legal steps and help you understand the implications and responsibilities of becoming a guardian or obtaining power of attorney.  3. Determine capacity: Determine if your loved one has the legal capacity to make decisions. If they are still capable of understanding and making informed choices, it may be possible to establish a power of attorney. However, if their cognitive abilities are significantly impaired, guardianship might be necessary.  4. File a petition: If guardianship is required, you'll likely need to file a petition with the appropriate court. The court will evaluate the individual's capacity and determine if guardianship is necessary. This process may involve providing medical evaluations and other supporting documents.  5. Court evaluation: A court may appoint an independent evaluator, such as a physician or psychologist, to assess the individual's mental capacity and determine if guardianship is appropriate. The court will consider the evaluator's report when making a decision.  6. Attend a hearing: Once the petition is filed, you may need to attend a court hearing. During this hearing, you'll present your case, explaining why guardianship is necessary and why you should be appointed as the guardian. Other interested parties, such as relatives or friends, may also have the opportunity to voice their opinions.  7. Obtain court order: If the court determines that guardianship is necessary and that you are a suitable guardian, they will issue a court order granting you legal authority to act on behalf of your loved one. This order outlines your responsibilities and the extent of your decision-making authority.  It's important to note that laws and procedures can vary by jurisdiction, so it's advisable to consult with a legal professional who can provide guidance specific to your situation. They can help ensure that you follow the necessary steps and fulfill the legal requirements to obtain guardianship or power of attorney for your loved one. |
